# Muscle and Systemic Molecular Responses to a Single Flywheel Based Iso-Inertial Training Session in Resistance-Trained Men

**DOI:** 10.3389/fphys.2019.00554

**Published:** 2019-05-09

**Authors:** Giosuè Annibalini, Serena Contarelli, Francesco Lucertini, Michele Guescini, Serena Maggio, Paola Ceccaroli, Marco Gervasi, Carlo Ferri Marini, Francesco Fardetti, Eugenio Grassi, Vilberto Stocchi, Elena Barbieri, Piero Benelli

**Affiliations:** ^1^Department of Biomolecular Sciences, Division of Exercise and Health Sciences, University of Urbino Carlo Bo, Urbino, Italy; ^2^Interuniversity Institute of Myology, Urbino, Italy

**Keywords:** iso-inertial exercise, inflammation, growth factors, extracellular vesicles, circulating miRNAs

## Abstract

Growing evidence points to the effectiveness of flywheel (FW) based iso-inertial resistance training in improving physical performance capacities. However, molecular adaptations induced by FW exercises are largely unknown. Eight resistance-trained men performed 5 sets of 10 maximal squats on a FW device. Muscle biopsies (fine needle aspiration technique) and blood samples were collected before (t0), and 2 h (t1) after FW exercise. Blood samples were additionally drawn after 24 h (t2) and 48 h (t3). Paired samples *t*-tests revealed significant increases, at t1, of mRNA expression of the genes involved in inflammation, in both muscle (*MCP-1, TNF-*α*, IL-6*) and peripheral blood mononuclear cells (*IkB-*α*, MCP-1*). Circulating extracellular vesicles (EVs) and EV-encapsulated miRNA levels (miR-206, miR-146a) significantly increased at t1 as well. Conversely, muscle mRNA level of genes associated with muscle growth/remodeling (*IGF-1Ea, cyclin D1, myogenin*) decreased at t1. One-way repeated measure ANOVAs, with Bonferroni corrected *post-hoc* pairwise comparisons, revealed significant increases in plasma concentrations of IL-6 (t1; t2; t3) and muscle creatine kinase (t1; t2), while IGF-1 significantly increased at t2 only. Our findings show that, even in experienced resistance trained individuals, a single FW training session modifies local and systemic markers involved in late structural remodeling and functional adaptation of skeletal muscle.

## Introduction

Since the first appearance of an effective training apparatus able to combat the neuromuscular dysfunctions that occur during space flights (Berg and Tesch, 1994), gravity independent resistance exercise devices have found wide terrestrial applications in the fields of sports (de Hoyo et al., 2015), injury prevention (Askling et al., 2003), and clinical rehabilitation (Greenwood et al., 2007).

Gravity independent devices employ a flywheel (FW), which is held by a fixed shaft anchored to a strap, to provide the resistance to muscular work. Basically, the concentric part of the movement starts to unwind the strap, thus, to rotate the FW. As soon as the concentric phase ends the strap starts rewinding, thus initiating the eccentric part of the movement. In the eccentric phase, the resistance is provided by the kinetic energy previously accumulated by the FW, therefore, the greater the effort expended in the concentric action, the more energy required to brake the FW with the eccentric action.

Although still hotly debated (Vicens-Bordas et al., 2018), mounting evidence (Maroto-Izquierdo et al., 2017) suggests that FW training may offer several advantages in terms of muscular strength and power gains compared to conventional gravity dependent resistance training. Indeed, unlike conventional resistance training exercises, which imply sub-optimal loading of the eccentric muscular actions (e.g., see Hollander et al., 2007) and yield to maximal effort only at the ‘sticking points' of the last repetitions of each set (van den Tillaar et al., 2014; Kompf and Arandjelović, 2016), the iso-inertial accommodating resistance modality of the FW devices allows maximal effort from the very first repetition and through the entire range of motion of the concentric muscular actions (Tesch et al., 2017). Moreover, by using FW devices a higher load than that of the concentric phase can be achieved with the eccentric action, namely “eccentric overload,” when the time of application of the breaking force needed to slow down the FW is intentionally reduced (for details, see: Berg and Tesch, 1994; Tesch et al., 2017). This makes it possible to perform eccentric exercise avoiding the difficulties associated with the complex technical optimization of a strictly eccentric regimen (i.e., third-party assistance, computer-guided electrically-powered devices, etc.).

The popularity of terrestrial FW devices stems from their potential capacity to emphasize eccentric muscle actions. Indeed, eccentric training offers unique neuromuscular, cardiorespiratory, and fatigue acute responses (Douglas et al., 2017), and chronic adaptations (Hedayatpour and Falla, 2015), which produce superior effects compared to conventional resistance exercise modalities (i.e., free weights, weight loaded systems, etc.). Eccentric actions, in fact, trigger subcellular damage to the contractile and structural components of skeletal muscle that induce both local and systemic inflammatory responses, which, in turn, stimulate muscle protein synthesis and satellite cell proliferation and differentiation (Chazaud, 2016).

Within skeletal muscle, the exercise-induced inflammatory response includes activation of the transcription factor nuclear factor-κB (NF-κB) and an increase in cytokines, such as interleukin-6 (IL-6), tumor necrosis factor-α (TNF-α), and monocyte chemoattractant protein-1 (MCP-1) (Peake et al., 2005). The initial proinflammatory response to muscle injury is required for all subsequent phases of inflammation that are part of the recovery process involving satellite cell activation and muscle regeneration (Tidball et al., 2014). In particular, the local production of insulin-like growth factor-1 (IGF-1) plays a crucial role in inflammation resolution and, together with myogenic regulatory factors (MRFs), controls for satellite cell activation during myogenesis and muscle regeneration (Schiaffino et al., 2013).

The systemic responses to eccentric exercise comprise leukocytes activation and serum increase in muscle damage [e.g., muscle creatine kinase (CKM)] and inflammation (e.g., IL-6, IL-1 and IL-1ra) markers (Vincent et al., 2014). The systemic response to exercise also affects circulating micro-RNAs (miRNAs) and, particularly, skeletal-muscle specific miRNAs (e.g., miR-1, miR-133b, miR-206, and miR-499) (Kirby and McCarthy, 2013). Notably, differential regulations of circulating miRNAs in response to eccentric vs. concentric exercise have been reported (Banzet et al., 2013). Moreover, miRNAs do appear to be useful as biomarkers for exercise-induced muscle damage (Uhlemann et al., 2014). The physiological implications of exercise-induced changes in circulating miRNA levels have yet to be fully understood (Kirby and McCarthy, 2013). One possible mechanism may involve the release of extracellular vesicles (EVs) carrying bio-active miRNAs by the active skeletal muscle thereby acting as a paracrine factor influencing the activity of other tissues (Guescini et al., 2017; Whitham et al., 2018). This is a very important issue because EV-miRNAs can be internalized within the target cell and regulate gene expression (Forterre et al., 2014; Guescini et al., 2017), whereas it is still unclear if the same could happen for free-miRNAs as well.

Few studies have investigated the acute responses of systemic markers of muscle damage and inflammation to a FW-based training session, in subjects not actively involved in resistance training (Carmona et al., 2015; Coratella et al., 2016). Moreover, the muscular local effects of FW exercise are still unknown. We hypothesized that, in resistance trained individuals, the FW exercise increases muscular and systemic inflammatory responses and muscle remodeling marker levels. Accordingly, the aim of this study was to characterize the molecular markers involved in inflammation and muscle remodeling in response to a single FW resistance training session.

## Materials and Methods

### Subjects

Eight recreationally resistance-trained healthy male subjects (age, 23.7 ± 2.8 years; height, 1.79 ± 0.09 m; body mass, 78 ± 8 kg; BMI, 24.4 ± 1.6 kg/m^2^; resistance training experience, 6.6 ± 2.4 years) were enrolled. Subjects were deemed as recreationally resistance-trained when they engaged in resistance exercise for general health-related purposes, 2–5 days per week in the 12-month period before the beginning of the study. Before enrolment, subjects completed a medical history questionnaire, underwent a physical examination performed by a sports medicine specialized physician, received oral and written explanations about the study design, and signed an informed consent form.

The study was approved by the Human Research Ethics Committee of the University of Urbino Carlo Bo and all the experimental procedures employed in the study conformed to the ethical considerations of the Declaration of Helsinki.

### Study Design

In this single-group study a pre-post intervention design was employed to assess the effect of a single FW resistance exercise session on the modulation of both muscular and systemic molecular markers of inflammation and muscle remodeling.

Participants undertook two familiarization sessions with the FW device and then underwent two testing sessions. Each of those four visits to the lab was separated by 1 week. Participants then reported to the lab two more times for further blood samplings. All sessions were carried out in the morning, and participants were instructed to maintain their dietary habits and to abstain from any exercise in the 4 days leading up to both testing sessions and in the 2 days following the last testing session.

**Figure 1:**
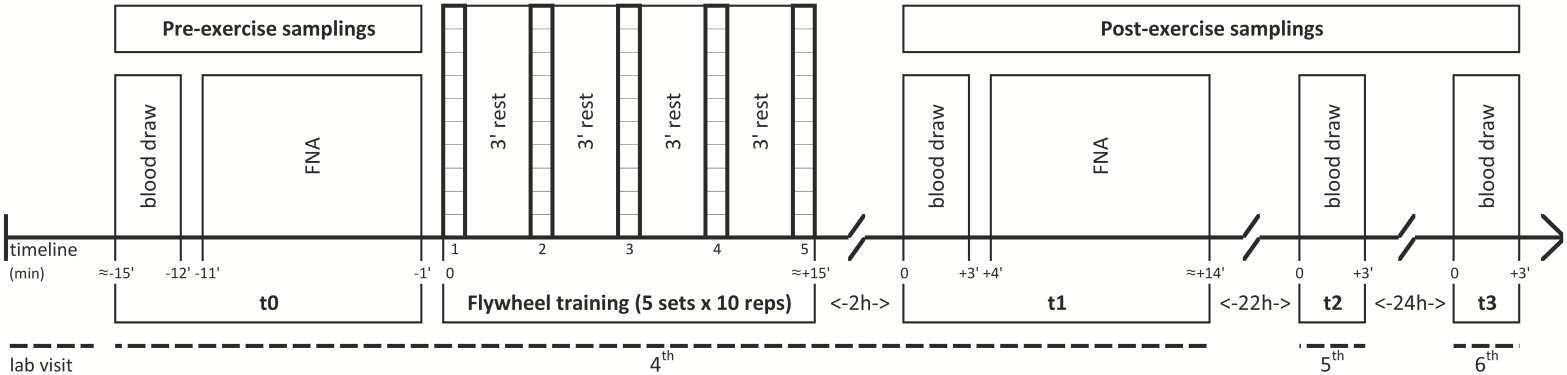
Experimental design of the flywheel training session (4^*th*^ visit) and of the following blood draws (5^*th*^ and 6^*th*^ visit). The time schedule of pre- (t0) and post-exercise (t1; t2; t3) samplings is shown below the timeline. FNA, fine needle aspiration.

Enrolment procedures were carried out before starting the exercise of the first familiarization session (1st visit), while anthropometric assessment was performed before the second one (2nd visit). Participants' peak iso-inertial power output was assessed in the first testing session (3rd visit), while in the second testing session (4th visit) participants undertook a FW resistance training routine at least 2 h after a light Mediterranean breakfast (60% carbohydrates, 20% proteins, 20% fats). Immediately before (t0) and 2 h after (t1) the training routine blood samples were drawn and muscle needle biopsy samples were collected. Blood samples were also drawn 24 h (t2) and 48 h (t3) after the end of the training routine (5 and 6th visit, respectively). See Figure 1 for details.

### Flywheel Training Session

Participants performed 5 sets of 10 maximal squats (with 3-min rest intervals in between) on a FW device (model *D11-Sport*, Desmotec S.r.l., Biella, BI, Italy) placed under the barbell of a Smith machine (model *Multipower Selection*, Technogym S.p.a., Cesena, FC, Italy). They wore a harness—fastened on both shoulders and waist—anchored to the strap of the FW device by means of a snap-hook placed between the thighs, just below the pubic region. The FW device was equipped with a height-adjustable, telescopic kit providing for a reference point (knee angle of 90°) for both the starting position and the end of the downward action of each repetition. According to the directions of the manufacturer, the length of the strap was set at its maximum length with the participant standing (knee angle of 180°) with his feet placed shoulder width apart and the strap wound until the participant reached the starting position. Within 3 s (in order to avoid unnecessary fatiguing isometric contractions), participants were asked to perform 10 reps, exerting as much force and speed as possible during each concentric action, while gripping (just outside the width of the shoulders) the barbell of the Smith machine (which was equipped with a barbell pad and placed high up on the back of the shoulders). The FW device was previously loaded with one or more disks providing the inertial resistance that yielded each participants' peak power output (which were obtained in the first testing session–see below for details), whereas the barbell of the Smith machine was unloaded. The Smith machine employed a counterbalanced system that allows the subject to perform the upward movement of the unloaded barbell with minimum effort and was used in the attempt to reduce the anterior-posterior movements of the body during the maximal squatting actions (see Video S1 in the Supplementary Material for set up and workout details).

### Assessment Procedures

#### Anthropometrics

Height (head in the Frankfurt plane; m) and weight (light clothes, without shoes; kg) were measured with the participant standing and used to calculate (kg/m^2^) body mass index (BMI).

#### Peak Iso-Inertial Power Output

A maximal power test was performed with the same devices and under the same exercise settings of the FW training session (see a previous paragraph for details). Adjunctively, the FW was equipped with a strain gauge placed between the snap-hook and the strap of the FW device, while the cable of a linear encoder was anchored to one side of the barbell of the Smith machine. Strain gauge and linear encoder data were simultaneously sampled at 100 Hz using a multichannel data acquisition system (model *APLab-DAQ*, APLab, Rome, RM, Italy).

Participants performed 6 sets of maximum 8 squats, with 5 min rest in-between, and were asked to always perform the concentric part of each squat at the maximal speed. The load placed on the FW was increased after each set by either adding one or more inertial disk(s) or substituting the inertial disk with one with greater mass (see Table 1 caption for further details). The participant was allowed to end the set before the completion of 8 reps when two consecutive reps yielded a power output lower than that recorded in one of the preceding reps. The power output of the concentric part of each squat was calculated as the product of the instantaneous speeds (in m·s^−1^) and loads (N). The highest power output of each rep was compared to those of the other reps of the same set and to those of the reps of the other sets performed, and the highest power output calculated was defined as the peak power output.

**Table 1:** Individual results of the maximal iso-inertial power output test.

**Participant ID**	**Peak power output (W)**	**Set (#)**	**Rep (#)**
1	1837.3	4	2
2	2049.6	2	4
3	1804.5	4	1
4	1685.0	2	4
5	2297.3	4	1
6	1756.6	2	1
7	1543.4	2	5
8	1627.6	2	7

*The “Set” and “Rep” columns display the number (#) of set, and the rep of that set, in which the participant reached the peak power output. Inertial loading details: Set#1, 1 disk size Large; Set#2, 2 disks size Large; Set#3, 1 disk size Medium + 1 one disk size Pro; Set#4, 1 disk size Large + 1 disk size Pro; Set#5, 2 disks size Large + 1 disk size Pro (according to the inertial set availability and increments provided by the manufacturer of the FW device)*.

#### Muscle Sampling

Skeletal muscle samples were obtained by fine needle aspiration (FNA) from the *vastus lateralis* muscle, as previously described elsewhere (Guescini et al., 2007). Briefly, a 22-gauge needle was inserted in the middle portion of the *vastus lateralis* muscle and kept firm. Each sample was collected by creating a constant vacuum using a 60 mL plastic syringe. The plunger of the syringe was maintained at its maximal extension for 2 min by a firm support placed between the barrel and the plunger flanges. At the end of the procedure, the needle was washed with 1 mL of RLT lysis buffer (Qiagen, Milan, MI, Italy) diluted 1:3 by diethyl pyrocarbonate treated water. Samples were then stored at −80°C until RNA extraction. As previously reported (Guescini et al., 2007), FNA is a safe and minimally invasive technique that does not require local anesthesia. A preliminary experiment performed to rule out potential artificial changes in mRNA expression due to the inherent sampling procedure showed that FNA, *per se*, did not change the expression of genes involved in the inflammatory response (see discussion and Figure S1 for details).

#### Blood Sampling

Venous blood samples (~15 mL per sample) were obtained from the antecubital vein and collected into BD Vacutainer® EDTA tubes (BD diagnostic preanalytical systems, Milan, MI, Italy). The venipunctures at the 4 timepoints were made alternatively in the right and left arm of the participant in order to avoid the potential effects of multiple needle sticks on systemic inflammatory responses. Blood samples were centrifuged at 1,000 g at 4°C for 15 min to obtain the plasma, which was stored at −80°C for later analysis.

### Muscle and Blood Sample Analyses

#### Multiplexed Tandem Real Time PCR Quantification of mRNA Expression

Total RNA of muscle FNA samples was extracted with RNeasy® Micro Kit (Qiagen, Milan, MI, Italy) and complementary DNA was synthesized using Sensiscript® Reverse Transcription Kit (Qiagen, Milan, MI, Italy) with random hexamers or an anchored oligo-dT primer (5′-AAGCAGTGGTATCAACGCAGAGTACT(30)NV-3′) where specified. Gene expression analysis was achieved by the Multiplexed Tandem Real time PCR strategy as reported by Guescini et al. (2007). Briefly, a preamplification step was performed with a mixture of specific primers (100 nM) for each target (see Table S1) using HotStart Taq DNA Polymerase (Qiagen, Milan, MI, Italy). To pre-amplify the *IGF-1* mRNA variants a reverse primer corresponding to the anchor sequence of the RT primers was used in combination with a common forward primer for the three isoforms (5′-CCTCCTCGCATCTCTTCTACCTG-3′). Real-time quantitative PCR was performed by mixing 2 μL of template from the first PCR and 10 μM of each specific internal primer (see Table S1) in an Applied Biosystems StepOnePlus™ Real Time PCR System using SYBR® Select Master Mix (Applied Biosystems, Monza, MB, Italy) or TaqMan® Universal PCR Master Mix (Applied Biosystems, Monza, MB, Italy) where specified. The mRNA expression data were normalized to the geometric mean of *GAPDH* and *B2M* reference genes. The sequences of primers used in real time PCR quantification are listed in Table S1.

#### ELISA Assays

Commercially available ELISA kits were used to determine the concentration of CKM (Cloud-Clone Corp SEA109Hu; Aurogene S.r.l., Roma, RM, Italy), IGF-1 (BioVendor RMEE20; Aurogene S.r.l., Roma, RM, Italy), and IL-6 (R&D system D6050; Space Import-Export S.r.l., Milan, MI, Italy). All samples were assayed in duplicate. The intra-assay coefficients of variation of the ELISA kits were: CKM, <10%; IGF-1, <5.8%; IL-6, <4.2%.

#### PBMC Isolation and Real Time PCR Quantification of mRNA Expression

PBMCs were isolated from EDTA blood by density gradient centrifugation on Lymphoprep^TM^ by Axis-Shield (Sentinel Diagnostic, Milan, MI, Italy), as described elsewhere (Annibalini et al., 2017). Briefly, 3.5 mL of blood samples were diluted with an equal volume of physiological saline and layered on 3 mL of Lymphoprep. The suspension was then centrifuged at 800 g for 20 min at room temperature, and the central ring containing the PBMC was carefully recuperated with a Pasteur pipette. PBMC were washed twice with an equal volume of PBS, centrifuged at 400 g for 10 min and finally lysed in 700 μL of Trizol. Total RNA was extracted and purified using the Omega Bio-Tek E.Z.N.A.^TM^ total RNA kit (VWR International, Milan, MI, Italy) according to the manufacturer's instructions. After DNA digestion with DNase I enzyme (Qiagen, Milan, MI, Italy) complementary DNA was synthesized from 1 μg of total RNA using Omniscript RT (Qiagen, Milan, MI, Italy) and random hexamers. Real-time quantitative PCR was performed with 2 μL of cDNA and 300 nM of each primer in an Applied Biosystems StepOnePlus™ Real Time PCR System using SYBR Select Master Mix (Applied Biosystems, Monza, MB, Italy). The sequences of primers used in real time RT-PCR quantification are listed in Table S1.

#### Extracellular Vesicles Isolation and miRNA Quantification

EV isolation was carried out as described in Guescini et al. (2015). In brief, 5 mL of plasma was first cleared by centrifugation for 15 min at 1,000 g to eliminate cell contamination. Supernatants were diluted with a same volume of PBS and further centrifuged for 20 min at 12,000 g and subsequently for 20 min at 18,000–20,000 g. The resulting supernatants were pelleted by ultracentrifugation at 110,000 g for 70 min. The EV pellets were washed in 13 mL PBS, pelleted again and resuspended in PBS for nanoparticle tracking assay before the lysis with 700 μL of Trizol for RNA extraction. The extraction of miRNA from the EV pellets was performed using the Total Exosome RNA & Protein Isolation Kit (Invitrogen, Milan, MI, Italy). Reverse transcription of RNA was performed using miRCURY LNA Universal RT microRNA PCR, Polyadenylation and cDNA synthesis kit II according to manufacturer's instructions (Exiqon, Milan, MI, Italy). Real-time quantitative PCR for the cDNA extracted from the EVs were performed in a StepOnePlus Real-Time PCR (Applied Biosystems, Monza, MB, Italy) using the ExiLENT SYBR® Green master mix and the miRCURY LNA miRNA PCR Assays (Exiqon, Milan, MI, Italy), specific for human miR-133b, miR-206, miR-126-3p, and miR-146a-5p.

### Statistical Analysis

Normality of distribution was assessed for each variable using the Shapiro-Wilk test. Non-normally distributed variables (i.e., relative mRNA and miRNA expression levels and CKM concentration) were log transformed. Separate one-way repeated measure ANOVAs were run on CKM, IGF-1, and IL-6 concentrations. The assumption of sphericity was met in each ANOVA (Mauchly's sphericity test was used). When significant main effects were found, Bonferroni corrected *post-hoc* pairwise comparisons were performed. Pre- vs. post-exercise comparisons of muscle FNA and PBMC gene expression data were performed using paired *t*-tests. Eta-squared (η^2^) and Cohen's effect size (ES) were determined for one-way repeated measure ANOVAs and paired *t*-tests, respectively. The level of statistical significance was set at *p* < 0.05. Statistical analyses were performed with SPSS (IBM SPSS Statistics for Windows v20.0, IBM Corp.).

## Results

The mRNA and miRNA expressions are reported as mean fold change (FC) and 95% confidence interval (CI) relative to pre-exercise (t0) data (which was set to 1). For each pairwise comparison of normally distributed variables (i.e., IL-6 and IGF-1), the mean difference (MD) and 95% CI were determined, whereas the median difference and the interquartile range were calculated for the non-normally distributed one (i.e., plasma CKM).

### Local Muscular Responses

#### Muscular Gene Expression Levels

**Figure 2:**
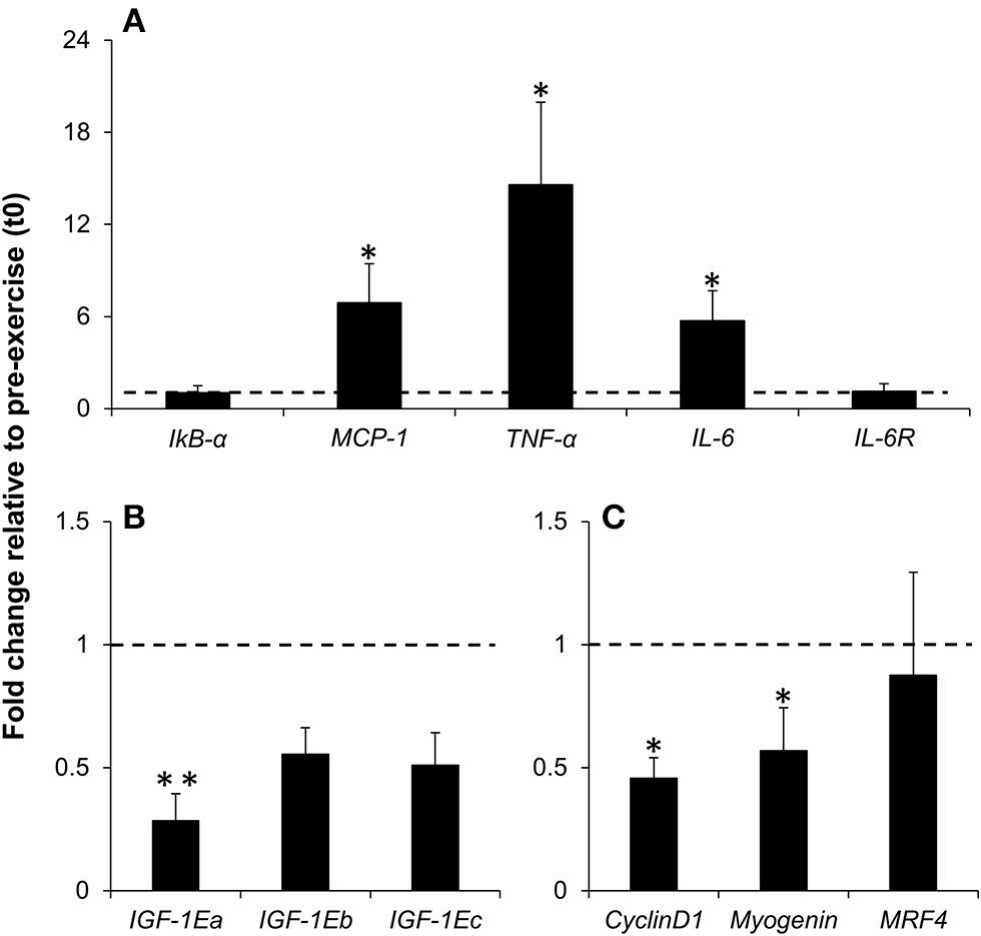
Fold change in *IkB*α*, MCP-1, TNF-*α*, IL-6, IL-6R* **(A)**, *IGF-1* isoforms **(B)** and *cyclin D1, myogenin* and *MRF4* **(C)** mRNA levels in muscle FNA samples obtained 2 h post-exercise (t1) compared to the pre-exercise (t0) (represented by the dotted line). Values are mean ± SE. *, significant difference from t0 (*p* < 0.05); **, highly significant difference from t0 (*p* < 0.01); *n* = 8.

The mRNA expression of *MCP-1* (FC = 6.89, 95% CI = 0.96 to 12.82, *p* = 0.030, ES = 0.96), *TNF-*α (FC = 14.60, 95% CI = 2.06 to 27.14, *p* = 0.047, ES = 0.85) and *IL-6* (FC = 5.72, 95% CI = 1.14 to 10.29, *p* = 0.047, ES = 0.85) increased at t1, while the mRNA expression of *IkB-*α (FC = 1.03, 95% CI = −0.07 to 2.13, *p* = 0.18, ES = −0.53) and *IL-6R* (FC = 1.13, 95% CI = 0.03 to 2.23, *p* = 0.237, ES = −0.46) did not change significantly over time (Figure 2A). The mRNA expression of *IGF-1Ea* decreased significantly at t1 (FC = 0.28, 95% CI = 0.03 to 0.54, *p* = 0.009, ES = −1.28), while *IGF-1Eb* (FC = 0.55, 95% CI = 0.30 to 0.80, *p* = 0.092, ES = −0.69) and *IGF-1Ec* (FC = 0.51, 95% CI = 0.20 to 0.82, *p* = 0.111, ES = −0.65) gene expression did not change significantly (Figure 2B). Gene expression of *cyclin D1* (FC = 0.45, 95% CI = 0.26 to 0.65, *p* = 0.022, ES = −1.04) and *myogenin* (FC = 0.57, 95% CI = 0.16 to 0.98, *p* = 0.034, ES = −0.93) decreased at t1, while the mRNA expression of *MRF4* did not change significantly over time (FC = 0.87, 95% CI = −0.10 to 1.85, *p* = 0.142, ES = −0.59) (Figure 2C).

### Systemic Responses

#### Plasma Inflammatory and Muscle-Remodeling Marker Levels

**Figure 3:**
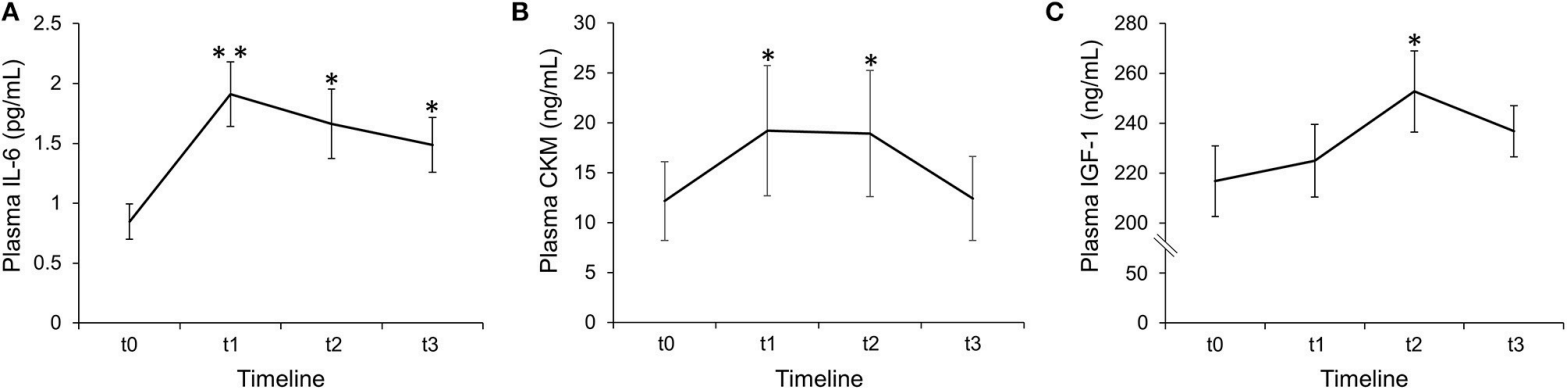
Quantification of IL-6 **(A)**, CKM **(B)**, and IGF-1 **(C)** plasma levels before (t0) and after (t1: 2 h; t2: 24 h; t3: 48 h) the flywheel training session. Values are mean ± SE. *, significant difference from t0 (*p* < 0.05); **, highly significant difference from t0 (*p* < 0.01); *n* = 8.

The ANOVA revealed a significant main effect of time for plasma level of IL-6 [*F*_(3,21)_ = 10.8, *p* = 0.001, η^2^ = 0.607], CKM [*F*_(3,21)_ = 7.58, *p* = 0.001, η^2^ = 0.520] and IGF-1 [*F*_(3,21)_ = 5.24, *p* = 0.007, η^2^ = 0.428]. *Post-hoc* pairwise comparisons revealed that IL-6 concentration increased significantly at t1 (*MD* = 1.06 pg/mL, 95% CI = 0.31 to 1.82, *p* = 0.008, ES = 1.81), t2 (*MD* = 0.81 pg/mL, 95% CI = 0.15 to 1.48, *p* = 0.018, ES = 1.57) and t3 (*MD* = 0.64 pg/mL, 95% CI = 0.14 to 1.14, *p* = 0.015, ES = 1.63) compared to t0 (Figure 3A). Plasma CKM significantly increased only at t1 (median difference = 5.11 ng/mL, interquartile range = 3.17–6.85, *p* = 0.013, ES = 1.66) and t2 (median difference = 4.36 ng/mL, interquartile range = 2.00–8.05, *p* = 0.016, ES = 1.61) compared to t0 (Figure 3B). IGF-1 plasma level significantly increased only at t2 (*MD* = 35.95 ng/mL, 95% CI = 0.15 to 71.75, *p* = 0.049, ES = 1.29) compared to t0 (Figure 3C). No significant differences were found between the other time points in any dependent variables.

#### PBMC Gene Expression Levels

**Figure 4:**
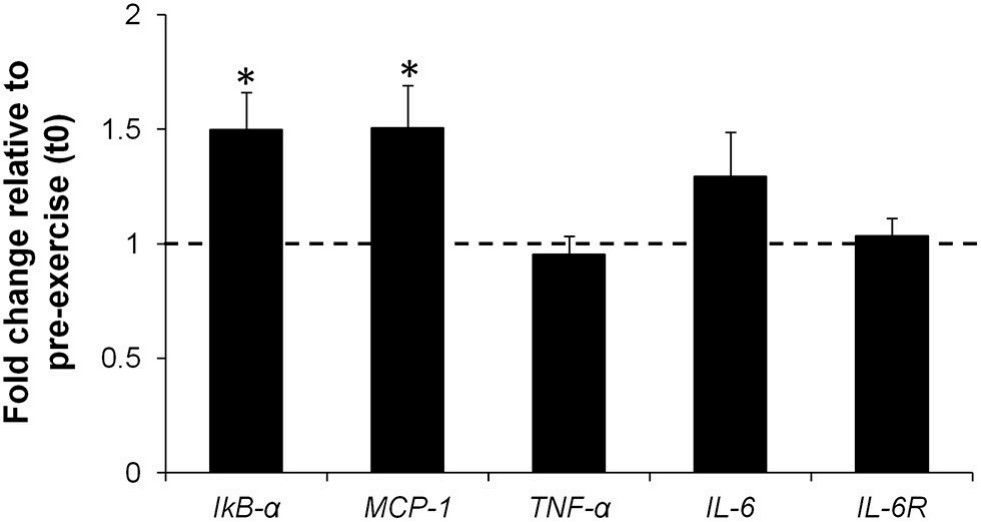
Fold change in *IkB-*α, *MCP-1, TNF-*α, *IL-6, IL-6R* mRNA levels in PBMC isolated 2 h post-exercise (t1) compared to pre-exercise (t0) (represented by the dotted line). Values are mean ± SE. *, significant difference from t0 (*p* < 0.05); *n* = 8.

Expression of *IkB-*α (FC = 1.50, 95% CI = 1.16 to 1.88, *p* = 0.017, ES = 1.11) and *MCP-1* (FC = 1.50, 95% CI = 1.07 to 1.94, *p* = 0.013, ES = 1.18) genes increased significantly at t1, while mRNA levels of *TNF-*α (FC = 0.95, 95% CI = 0.77 to 1.14, *p* = 0.432, ES = −0.29)*, IL-6* (FC = 1.29, 95% CI = 0.84 to 1.74, *p* = 0.216, ES = 0.48), and *IL-6R* (FC = 1.03, 95% CI = 0.86 to 1.21, *p* = 0.885, ES = 0.05) did not change significantly (Figure 4).

#### Plasma EVs and EV-Encapsulated miRNA Levels

**Figure 5:**
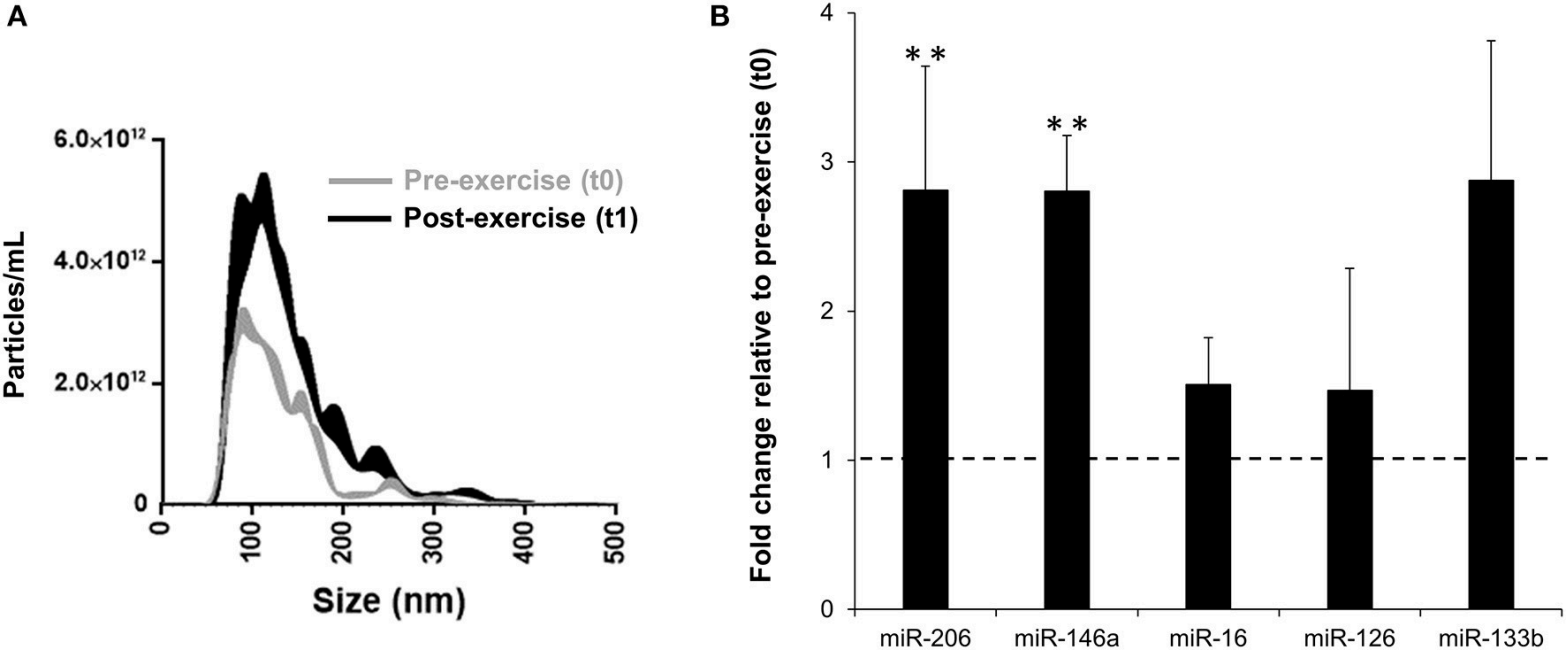
Post-exercise (t1) vs. pre-exercise (t0) nanoparticle tracking assay of circulating EVs **(A)** and fold change in miRNA levels in circulating EVs **(B)**. Pre-exercise values within **(B)** are represented by the dotted line. Values are mean ± SE. **, highly significant difference from t0 (*p* < 0.01); *n* = 8.

A 2-fold increase of circulating EVs was evident at t1 (Figure 5A). EV-encapsulated miR-206 (FC = 2.81, 95% CI = 0.89 to 4.73, *p* = 0.010, ES = 1.25) and miR-146a (FC = 2.80, 95% CI = 0.86 to 4.75, *p* = 0.008, ES = 1.28) levels increased significantly at t1 (Figure 5B), while miR-16 (FC = 1.51, 95% CI = 0.63 to 2.38, *p* = 0.569, ES = 0.21), miR-126 (FC = 1.47, 95% CI = 0.74 to 2.20, *p* = 0.442, ES = 0.29) and miR-133b (FC = 2.88, 95% CI = 0.69 to 5.07, *p* = 0.189, ES = 0.51) expression did not change significantly.

## Discussion

Despite its growing popularity among competitive and non-competitive athletes, and researchers, the exercise-induced molecular responses to a single FW iso-inertial resistance training session are largely unknown. The main finding of the present descriptive study is that FW exercise acutely affects the local and systemic markers involved in late structural remodeling and functional adaptation of skeletal muscle. Indeed, increased plasma concentrations of the muscle damage marker CKM and the multifunctional cytokine IL-6 were found 2 h after exercise and during the 48 h recovery period, along with acute increases in *IkB-*α and *MCP-1* mRNAs in PBMC. Using muscle FNA we also demonstrated that *MCP-1, TNF-*α, and *IL-6* gene expression increased 2 h after the FW training session.

Two recent studies dealing with the acute effects of FW exercise showed an increase in muscle damage (i.e., CKM and fiber type-specific sarcomere proteins in blood) and inflammation (i.e., IL-6) markers in young males not actively involved in resistance training (Carmona et al., 2015; Coratella et al., 2016). The results of the present study confirm and extend these findings by demonstrating, for the first time in resistance trained subjects, that FW exercise induces a transient increase in local and circulating molecular markers of inflammation and muscle damage. To the best of our knowledge, only three studies have investigated the response of some of the parameters we examined at time points similar to those used in the present study in resistance trained subjects squatting in a “traditional” manner (i.e., using a loaded barbell or a smith machine) for a similar number of sets and reps [6 sets of up 10 reps at 80% of 1 repetition maximum (RM) with 3 min rest in (Yamamoto et al., 2008); 4 sets of 10 reps at 70% of 1RM with 3 min rest in (Oliver et al., 2016); 3 sets of 8 reps at 80% of 1RM with 5 min rest in (Gonzalez-Badillo et al., 2016)]. With regards to CKM and IL-6, the magnitude and duration of the post-training increases found in those studies were comparable to the results of the present investigation, indicating a mild muscle damage and inflammation response (Paulsen et al., 2012) regardless of the device employed for the exercise.

The early muscular inflammatory and immune responses are crucial processes that discard tissue debris and allow tissue repair (Chazaud, 2016). Accordingly, interfering with the initial inflammatory response by anti-inflammatory medication (Urso, 2013) or by disrupting monocyte recruitment (in MCP-1-deficient mice) (Shireman et al., 2007) delays or even impedes skeletal muscle regeneration. The local muscle proinflammatory response is accompanied by a systemic response that also includes the activation of leukocytes (Peake et al., 1985). In this regard, in the current study we found that FW training acutely increases *IkB-*α and *MCP-1* mRNA expression in PBMC. This increase is consistent with the results of other studies, showing that acute leukocyte activation after a single eccentric exercise plays a central role in ongoing tissue surveillance and muscle regeneration (García-López et al., 2007; Hyldahl et al., 2011).

After the FW training, we also observed an acute increase in EVs in the bloodstream and EV-encapsulated miR-206 and miR-146a. Recent studies have shown that muscle acutely contributes to the exercise-induced secretion of EVs after exercise (Guescini et al., 2015; Whitham et al., 2018). Moreover, EVs liberated by exercise are involved in cell-cell communication and, like hormones and cytokines, mediate gene expression in the target cells (Forterre et al., 2014; Guescini et al., 2017). Data from the present study on EV-encapsulated miR-206 and miR-146a upregulation after the FW training session are in line with those obtained by Baggish et al. (2011). The miR-206, along with other “myomiRNAs” such as miR-1 and miR-133b, are specific to or enriched in the skeletal muscle tissue and play important roles in the regulation of muscle development and differentiation (Kirby and McCarthy, 2013). Hence, the reported increase in circulating EV-incapsulated mir-206 suggests that muscle contributes at least in part to the higher levels of circulating EVs following the exercise bout (Guescini et al., 2015). Many of the miRNAs altered by exercise are known to play mechanistic roles in the inflammatory processes (Baggish et al., 2011). Among these, miR-146a plays a key role in both the innate and adaptive immune response by negatively regulating TLR4 signaling and NF-κB-induced proinflammatory cytokine and chemokine expression (Williams et al., 2008; Magilnick et al., 2017). In this study, an increase in EV-encapsulated miR-146a was found after the FW training session. The EV-encapsulated miR-146a might be involved in the regulation of inflammatory responses induced by the FW exercise (Baggish et al., 2011). Moreover, these results are consistent with those of recent studies demonstrating the potential relevance of exercise-derived EVs as markers of muscle function and exercise adaptation (Guescini et al., 2015; Whitham et al., 2018).

The exercise-induced muscle damage is also followed by coordinated expression of growth factors and MRFs characterizing the skeletal muscle repair process (Chazaud, 2016). In this study muscle mRNA expression of the *IGF-1Ea* isoform, *cyclin D1*, and *myogenin* decreased 2 h after the FW training session. No changes from resting levels were observed for the plasma level of IGF-1 2 h after the FW training, while the IGF-1 concentration significantly increased after 24 h. The influence of IGF-1 and MRFs in mediating some of the beneficial aspects of exercise has been an area of intense interest, particularly with regard to post-exercise recovery and muscle tissue remodeling mechanisms (Nindl and Pierce, 2010; Stewart and Pell, 2010). However, conflicting results have been reported on the effect of exercise on skeletal muscle *IGF-1* mRNAs (Bamman et al., 2001; Bickel et al., 2003; Psilander et al., 2003; Coffey et al., 2009). Our data are in agreement with those obtained by Coffey et al. (2009), who showed that during the early phases of muscle recovery after resistance exercise, there is a temporary suppression of the molecular processes that promote protein synthesis (e.g., IGF-1), myoblast proliferation (e.g., cyclin D1), and maintenance of muscle mass (e.g., myogenin). Indeed, in the present study, the exercise-induced increase in circulating IGF-1 occurred only 24 h after the FW training session. Other researchers have focused on changes in systemic IGF-1 levels following a single resistance exercise session, reporting either no changes (Kraemer et al., 2003; Dalbo et al., 2011) or slight increases (Gonzalez-Badillo et al., 2016). However, comparing the changes in serum IGF-1 across different types of resistance exercises is difficult since the magnitude of IGF-1 elevation is not only dependent on exercise selection, intensity, and volume, but also on gender, nutritional state, and other individual parameters (Nindl et al., 2011). Future studies, which include additional muscle tissue samplings beyond 2 h after exercise and the quantification of local IGF-1 protein production (e.g., in the interstitial fluid; Berg et al., 2006), may better clarify the acute response of IGF-1 to a single FW training session.

One important issue when assessing acute muscular responses to exercise is whether a prior muscle biopsy is a procedure that itself contributes to changes in gene expression (Friedmann-Bette et al., 2011; Van Thienen et al., 2014; Boman et al., 2015). Indeed, Friedmann-Bette *et al*. showed similar changes in the expression of *IL-6* and *IGF-1* genes within muscle in response to squatting exercise compared to the control condition that only involved multiple muscle biopsies without exercise (Friedmann-Bette et al., 2011). However, this does not represent an actual limitation here because FNA (Guescini et al., 2007), which is less invasive than either microbiopsy or Bergström needle techniques, was adopted in the present study with the aim of eliminating artifacts potentially attributable to the biopsy *per se*. This critical point was addressed in a preliminary experiment, which did not show any difference in the expression of genes involved in the inflammatory response when two FNA muscle samplings were performed in the same leg (Figure S1). We believe that we can therefore rule out artificial changes in mRNA expression inherently due to the sampling procedure.

In conclusion, the findings of the present study show that a single FW based iso-inertial training session seems to induce muscle microlesions and increases pro-inflammatory gene expression in resistance-trained men. These data also suggest an earlier activation of the processes related to muscular injuries compared to those involved in muscle growth and repair. Therefore, FW based iso-inertial exercise seems to induce early muscle and systemic molecular adaptations, even in trained individuals, that have the potential to induce a late hypertrophic response.

A question that remains unanswered is whether there is a difference between the transient inflammatory and muscle damage responses when exercise is performed with a FW-equipped device or with a conventional gravity dependent device. Although conducting comparative studies could be challenging because it is difficult to properly match the intensities of two inherently different resistance exercise stimuli, future experimental studies are needed to assess both the transient and adaptive responses to the FW exercise in terms of muscle damage and local and systemic inflammation.

## Data Availability

The datasets generated for this study can be found in the FigShare repository (https://doi.org/10.6084/m9.figshare.7117796.v5).

## Ethics Statement

This study was carried out in accordance with the recommendations on human research from the Ethics Committee of the University of Urbino Carlo Bo, with written informed consent from all subjects. All subjects gave written informed consent in accordance with the Declaration of Helsinki. The protocol was approved by the Human Research Ethics Committee of the University of Urbino Carlo Bo.

## Author Contributions

GA, SC, and FL conceived and designed the study, analyzed the data and drafted the manuscript. FL, MGe, CFM, EG, and PB designed, prescribed and supervised the exercise sessions and contributed to the drafting of the manuscript. FF, GA, and SC collected, processed and stored the biological samples. GA, SC, and EB performed the RT-PCR experiments and immunoassays. MGu, SM, and PC performed the extracellular vesicle isolations and miRNA quantifications and contributed to the drafting of the manuscript. VS, EB, and PB provided overall direction to the project and revised the manuscript. All authors contributed to manuscript revision, read and approved the submitted version.

### Conflict of Interest Statement

The authors declare that the research was conducted in the absence of any commercial or financial relationships that could be construed as a potential conflict of interest.
