# Supplementary material for: Muscle and Systemic Molecular Responses to a Single Flywheel Based Iso-Inertial Training Session in Resistance-Trained Men
Source: Front Physiol. 2019 May 9;10:554. doi: 10.3389/fphys.2019.00554 (PMC6521220; doi:10.3389/fphys.2019.00554)
Supplement: Supplementary file 1 [file Data_Sheet_1.PDF]

Table S1. List of the oligonucleotides used in this study.

**Oligonucleotide name**	**sequence 5'→ 3'**	Experiment
IkB-α Fw1	ACGAGCAGATGGTCAAGGAG	Multiplexed preamplification step
IkB-α Fw2	GGTGCTGATGTCAATGCTCA	SYBR Green real-time RT-PCR
IkB-α Rw	ACACCAGGTCAGGATTTTGC	
MCP-1 Fw1	CGCCTCCAGCATGAAAGTCT	Multiplexed preamplification step
MCP-1 Fw2	AGTCTCTGCCGCCCTTCT	SYBR Green real-time RT-PCR
MCP-1 Rw	GTGACTGGGGCATTGATTG	
TNF-α Fw1	CTGCTGCACTTTGGAGTGAT	Multiplexed preamplification step
TNF-α Fw2	GACAAGCCTGTAGCCCATGT	SYBR Green real-time RT-PCR
TNF-α Rw	TCTCAGCTCCACGCCATT	
IL-6 Rw1	GCTTGTTCCTCACTACTCTC	Multiplexed preamplification step
IL-6 Fw2	GGTACATCCTCGACGGCATCT	SYBR Green real-time RT-PCR
IL-6 Rw	GTGCCTCTTTGCTGCTTTCAC	
IL-6R Fw1	AAGACCCCCACTCCTGGAACT	Multiplexed preamplification step
IL-6R Fw2	GACATTCACAACATGGATGGTC	SYBR Green real-time RT-PCR
IL-6R Rw	CTTGCCCGAACTCCTCCT	
Cyclin D1 Rw1	TCTGGCATTTTGGAGAGGAAGTG	Multiplexed preamplification step
Cyclin D1 Fw2	CCGAGAAGCTGTGCATCTACAC	SYBR Green real-time RT-PCR
Cyclin D1 Rw	AGGTTCCACTTGAGCTTGTTCAC	
Myogenin Fw1	GCAGTGCCATCCAGTACATC	Multiplexed preamplification step
Myogenin Fw2	GAGTTCAGCGCCAACCCA	SYBR Green real-time RT-PCR
Myogenin Rw	AGGTTGTGGGCATCTGTAGG	
MRF4 Fw1	CTGGATCAGCAGGAGAAGATG	Multiplexed preamplification step
MRF4 Fw2	GACCCCTTCAGCTACAGACC	SYBR Green real-time RT-PCR
MRF4 Rw	ATGATCGGAAACACTTGGCC	
B2M Fw1	TGACTTTGTCACAGCCCAAG	Multiplexed preamplification step
B2M Fw2	GATCGAGACATGTAAGCAGC	SYBR Green real-time RT-PCR
B2M Rw	CAAACATGGAGACAGCACTC	
IGF-1 common Fw	CCTCCTCGCATCTCTTCTACCTG	Multiplexed preamplification step
IGF-1Ea	Hs01547657_m1	TaqMan real-time RT-PCR
IGF-1Eb Fw	CTACCAACAAGAACACGAAGT	
IGF-1Eb Fw	CTACCAACAAGAACACGAAGT	TaqMan real-time RT-PCR
IGF-1Eb Probe	AAGGAAAGGTTGGCCAAA	
IGF-1Ec Fw	GCCCCCATCTACCAACAAGAACAC	
IGF-1Ec Rw	TCCCTCTACTTGCGTTCTTCAAA	TaqMan real-time RT-PCR
IGF-1Ec Probe	AGAGAAGGAAAGGAAGTA	
GAPDH1	AATCAAGTGGGGCGATGCT	Multiplexed preamplification step
GAPDH2	Hs03929097_g1	TaqMan real-time RT-PCR

Note: *IkB-α*, nuclear factor of kappa light polypeptide gene enhancer in B-cells inhibitor alpha; *MCP-1*, monocyte chemotactic protein 1; *TNF-α*, tumor necrosis factor-alpha; *IL-6*, interleukin-6; *IL-6R*, interleukin-6 receptor; *MRF4*, myogenic regulator factor-4; *B2M*, beta 2 microglobulin; *IGF-1*, insulin-like growth factor-1; *GAPDH*, gliceraldeyde-3-phosphate dehydrogenase.
