# Supplementary figures and images for: Muscle and Systemic Molecular Responses to a Single Flywheel Based Iso-Inertial Training Session in Resistance-Trained Men
Source: Front Physiol. 2019 May 9;10:554. doi: 10.3389/fphys.2019.00554 (PMC6521220; doi:10.3389/fphys.2019.00554)

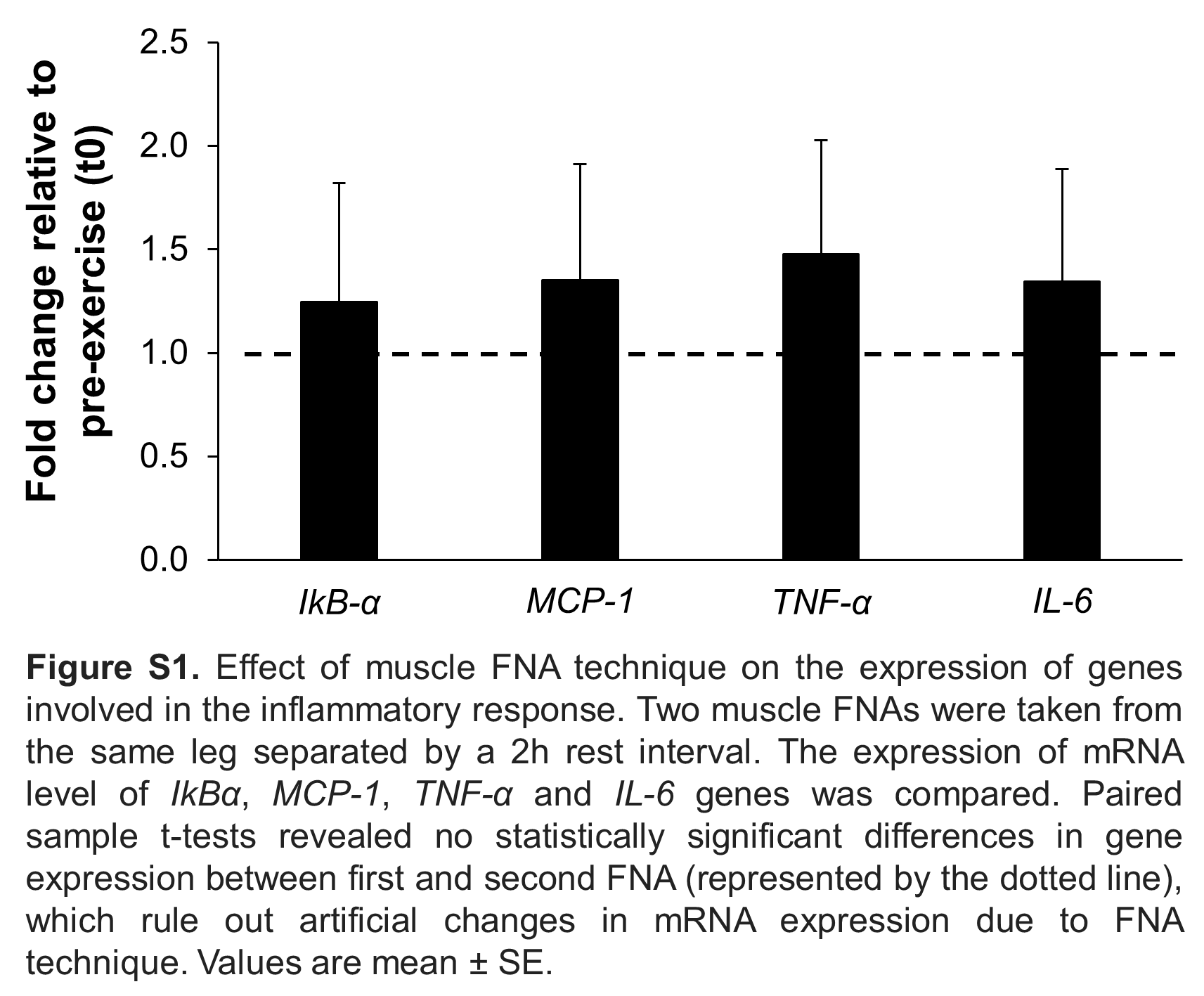

Supplement: Supplementary file 2 [file Image_1.TIF]
